# Guidelines for the role of autophagy in drug delivery vectors uptake pathways

**DOI:** 10.1016/j.heliyon.2024.e30238

**Published:** 2024-04-24

**Authors:** Moataz Dowaidar

**Affiliations:** a Bioengineering Department, King Fahd University of Petroleum and Minerals (KFUPM), Dhahran, 31261, Saudi Arabia; b Interdisciplinary Research Center for Hydrogen Technologies and Carbon Management, King Fahd University of Petroleum and Minerals (KFUPM), Dhahran, 31261, Saudi Arabia; c Biosystems and Machines Research Center, King Fahd University of Petroleum and Minerals (KFUPM), Dhahran, 31261, Saudi Arabia

**Keywords:** Autophagy, Drug delivery, Vectors, Uptake pathways, Nanocarriers, Nano-delivery Systems, Targeted Therapy, Cellular Trafficking, Nanotechnology, Organelle Specificity, Therapeutic Nanoparticles, Extracellular Vesicles, Selective Autophagy

## Abstract

The process of autophagy refers to the intracellular absorption of cytoplasm (such as proteins, nucleic acids, tiny molecules, complete organelles, and so on) into the lysosome, followed by the breakdown of that cytoplasm. The majority of cellular proteins are degraded by a process called autophagy, which is both a naturally occurring activity and one that may be induced by cellular stress. Autophagy is a system that can save cells' integrity in stressful situations by restoring metabolic basics and getting rid of subcellular junk. This happens as a component of an endurance response. This mechanism may have an effect on disease, in addition to its contribution to the homeostasis of individual cells and tissues as well as the control of development in higher species. The main aim of this study is to discuss the guidelines for the role of autophagy in drug delivery vector uptake pathways. In this paper, we discuss the meaning and concept of autophagy, the mechanism of autophagy, the role of autophagy in drug delivery vectors, autophagy-modulating drugs, nanostructures for delivery systems of autophagy modulators, etc. Later in this paper, we talk about how to deliver chemotherapeutics, siRNA, and autophagy inducers and inhibitors. We also talk about how hard it is to make a drug delivery system that takes nanocarriers' roles as autophagy modulators into account.

## 1 Introduction

Autophagy, an important cellular homeostasis mechanism, has become a major player in nanodelivery, providing new ways to make drug delivery systems more effective and selective. By using the autophagic process, scientists can create nanoparticle systems that work with the autophagic machinery in a specific way. This lets therapeutic agents enter cells in a controlled and targeted way. Nanoparticles can be made to avoid lysosomal degradation, which is a key part of the autophagic pathway. This makes it easier for drugs to get to specific targets inside cells [1]. Furthermore, the modulation of autophagy can influence the therapeutic outcomes of nanomedicine. Cancer therapy uses nanoparticles to change autophagic responses to make treatment more effective [2]. Autophagy has two roles: it can either help cells stay alive or drive them to die. For instance, nanoparticles that induce autophagy can enhance the therapeutic targeting of cancer cells, while those that inhibit autophagy can prevent the development of drug resistance [3]. Creating nanoparticles that can release their payload in response to autophagic signals is also linked to nanodelivery [4]. This makes it possible for a coordinated therapeutic response. This approach not only enhances the precision of drug delivery but also minimizes off-target effects, showcasing the potential of integrating autophagy with nanotechnology to create more effective and safer therapeutic strategies. Putting autophagy and nanodelivery together is a new step forward in creating advanced drug delivery systems. It opens up new ways to make therapeutic interventions more specific, effective, and safe [5].

Autophagy keeps up with cell homeostasis in neurodegenerative and harmful conditions by reusing misfolded proteins and damaged organelles. It is comprised of a few fastidiously controlled and regulated exercises (for example, lysosomal combination, obliteration, lengthening, nucleation, and inception) [1]. Numerous studies conducted over the course of the last two decades have shed light on the significance of autophagy in human illnesses. Altering the activity of autophagy by targeting certain regulatory [2] variables has the potential to influence disease processes [3]. Autophagy is a fundamental evolutionary catabolic process that is responsible for the digestion of cytoplasmic components. Activation of autophagy in malignancies indicates oncogenic and tumour-suppressing properties, whereas downregulation of autophagy in malignancies indicates the opposite. As a result, essential autophagy targets need to be discovered so that new treatments may be developed [4].

One physiological and dynamic method for maintaining the equilibrium of digestion is through autophagy. It depends on the creation of two-layer film vesicles, or lysosomes, which hold parts inside cells like organelles that are broken or maturing and extra or old proteins. Microscopic organisms are thought to be the wellspring of autophagy, which has physiological importance. It is frequently accepted that basal autophagy provides a system for cell perseverance during times of supplement shortage. This is because the breakdown items are once again introduced into the metabolic and biosynthetic cycles in the wake of being liberated from lysosomes. In addition, autophagy protects cells from outside stresses by getting rid of harmful and damaged parts and items. These outside stresses include oxidative pressure (operating system), bacterial or viral infection, and endoplasmic reticulum stress (trama centers). This is accomplished by getting the cell free from destructive and harmed items as well as cell parts. Autophagy, in any case, influences malignant growth improvement in two ways [5].

According to one example, it stops the harmful growth of oncogenic flagging particles that cause chromosomal damage that leads to cancer. This stops the start, growth, and treatment of growth. Besides, it advances the development of cancer. Biomolecule reusing that is interrupted by autophagy is a common way for disease cells to get the energy they need during digestion and deal with the stresses of their environment. This training energizes cancer development and hostility. Focusing on autophagy straightforwardly is one potential therapy system for disease since malignant growth cells rely more on it than typical tissues [6] For a variety of different reasons, autophagy is an essential component in the maintenance of an organism's fitness. Constitutive autophagic responses break down byproducts of normal cellular metabolism that could be harmful to cells if they build up [7]. These products include damaged mitochondria and redox-active protein aggregates [8]. It was found that cells that react to disturbances in their intracellular or extracellular homeostasis exhibit inducible autophagic responses, which improve the survival of the cells. Stopping autophagy, either through drugs or genetics, often causes cells that are fighting infections and metabolic, physical, and chemical stresses to die too soon [9].

This shows that autophagy is an important part of the process of adapting to stress. In addition, autophagy has a close relationship with the cell-extrinsic circuitries that are responsible for the maintenance of homeostasis and the promotion of healthy aging at the level of the entire organism. Autophagic responses in tissues like the liver, skeletal muscle, and other tissues are thought to play a part in the positive effects of active work on glucose digestion throughout the body [10]. This is related to the fact that some types of chemotherapy and radiation treatment kill harmful cells, which sets off autophagy. This sends out warning signals and eventually starts a reaction that is not responsive to treatment [11]. Under certain pathophysiological conditions, autophagy may intercede cytotoxic impacts, but the expression “autophagic cell demise” ought to be utilized cautiously. Yet, the expression “autophagic cell demise” isn't one that should be utilized [12].

## 2 Autophagy

**Fig. 1:**
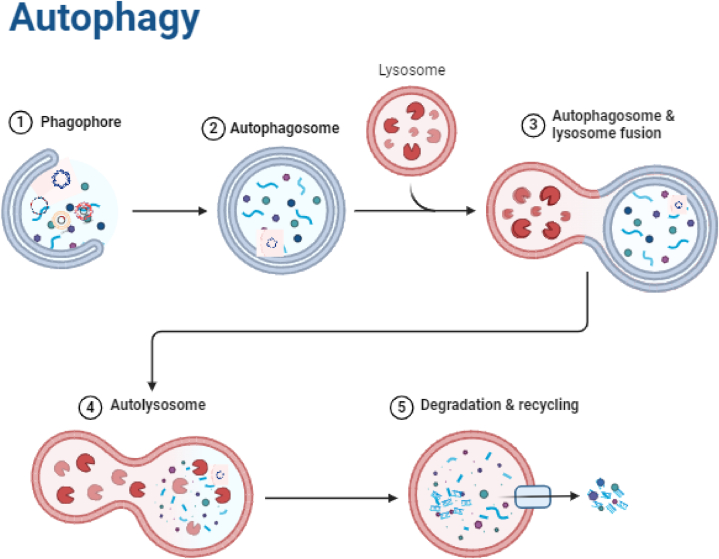
Process of autophagy (Created with BioRender.com**).**

In order to protect genomic integrity, help with assimilation, and support cell survival, autophagy stops the breakdown of cytoplasmic macromolecules. It is an important, universal, formatively moderated, catabolic, and self-degradative cycle. The Greek expressions auto, which signifies “self,” and phagy, which means to eat, are the beginning of the word autophagy [13,14]. A characteristic administrative system accomplishes housekeeping tasks like clearing up totaled or misfolded proteins, damaged organelles, and proteins, all while keeping up with sound synthetics in the body and disposing of hurtful ones [15,16], as well as substances that can cause malignant growth [17]. Lysosomal corruption is additionally used to dispose of unfamiliar infections [18]. Autophagy has been connected to various physiological cycles, for example, the end of exogenous side effects and outside substances to protect homeostasis outlines of autophagy process is shown in Fig. 1. Nonetheless, disturbing this system's regular harmony could have hindering impacts [19].

As the essential detoxification process in the body, autophagy can possibly forestall or treat disease by dispensing with unsafe cells and disposing of inner or outer toxins, advancing the development of solid cells. On the other hand, autophagy may play two roles in the growth of cancer cells: it may help with metastasis, growth repeat, and protecting developing cells from disease therapy (chemoresistance and radioresistance) [20]. Infections communicate with a range of exercises inside their host cells to endure, in light of the fact that they are compulsory intracellular parasites. These cycles incorporate immunological reactions, cell transport, and digestion [21]. Besides, autophagy is a significant system in the cell material corruption process and is embroiled in both natural and versatile safe reactions [22]. Moreover, in BV2 microglial cells, autophagy manages the phosphorylation of p38 and ERK1/2 MAPKs. The development of nitric oxide relies on this phosphorylation. Along these lines, it might decrease neurotoxicity and influence how microglia enact neuronal cells. Another thing it can do is stop the support of harmful go-betweens in BV2 microglial cells [23]. This keeps these cells safe from the neuronal cell passing that LPS and synuclein promote.

Specific and non-specific autophagy are two unique cycles. During specific autophagy, certain receptors can tell the freight apart, which lets the autophagosome find it, lock it up, and destroy it [24]. Conversely, the lysosome stalls all materials in vague autophagy in a way that isn't well defined for any certain thing. Besides, it is recognized that autophagy can happen in two unique ways: constitutively and responsively, or in light of an outside boost. The last option has been concentrated on exhaustively and is known to accelerate neurite rebuilding by creating cerebrums [25], proposing that it very well may be expected at each phase of mental health. On the other hand, constitutive autophagy has not drawn a lot of interest. Researchers [26] found that creatures without the autophagy proteins Atg59 and Atg710 had raised neurodegeneration, demonstrating a basic job for autophagy in the physiological elements of the body. Autophagy can be started by a number of things, including not having enough amino acids and chemicals in the diet, proteins that aren't growing properly, or microbes that are in the body. Both interior and exterior triggers can start autophagy, either at the point of obliteration or as a maintenance component [27].

Lack of amino acids, stress, and sudden drops in chemicals or trophic elements (for example, sex-based contrasts) [[28], [29]] can all set off autophagy. So, a lack of lipids and poor cholesterol transport inside cells This disease is caused by pathogenic microorganisms, protein fragments, and [30]. Neurotoxin (1-methyl-4-phenylpyridinium) can start the autophagy process, but two types of PI3K inhibitors can't stop it. They can only stop the process that starts when mitochondria don't have enough food, though. These two boosts are instances of how they can change autophagic capability and cause different morphological impacts [31]. Autophagy was discovered a long time ago, yet its sub-atomic instruments were not completely perceived until the last part of the nineties. This was made practical by transformations in autophagy-related genes found during a yeast hereditary screening. Something like thirty autophagy genes (Atgs) have been recognized in yeast, and the greater part of these have homologs in human cells. A wide range of sub-atomic pathways have been utilized to concentrate on the fundamental cycles that underlie autophagy. To set off autophagy, a few flagging pathways concentrate on two protein buildings. The PI3KC3–C1 lipid kinase complex is class III phosphatidylinositol 3-kinase complex I, which is, well, a lipid kinase complex. The ULK1 protein kinase complex is very interesting [32].

**Fig. 2:**
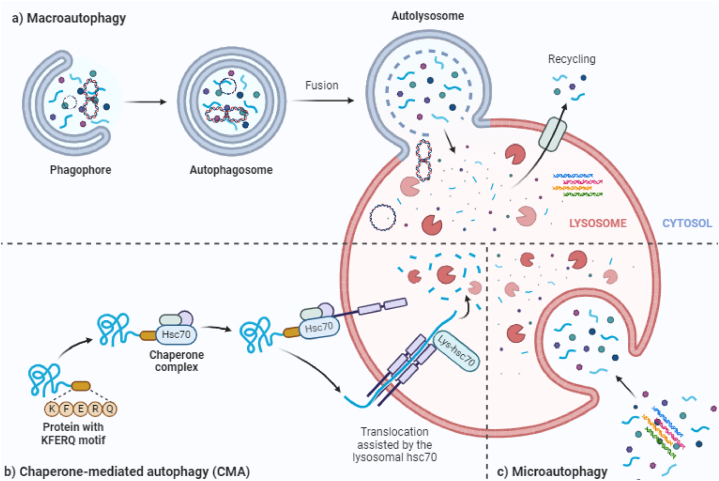
Autophagy may take place on three different scales: macro, micro, and chaperone-mediated (Created with BioRender.com**).**

It has additionally been shown that RNA-related exercises, including up until now unseen autophagy controllers, are associated with this cycle [33]. Also, it is known that the pressure in the emergency room sets off upstream signaling pathways using flagging atoms like Advantage/ATF4, IRE1, ATF6, and Ca2+. These pathways are similar to both autophagy and apoptosis. Regardless of the way that these pathways are shared by the two cell death components, this is the situation. The subtleties of these pathways will shed light on the different kinds of autophagy and the few intermediates included. Three particular types of autophagy are portrayed in Fig. 2: chaperone-mediated autophagy (CMA), microautophagy, and macroautophagy.

## 3 Autophagy uptake mechanisms

Autophagy is the most widely recognized system for moving cytoplasmic or extracellular materials to a creature's lysosomes, or the vacuoles of plant or yeast cells [34]. It is very easy to manage the area, timing, and force of autophagosome creation and ensuing development. Phosphoinositide-limiting protein HS1BP3 is an autophagosome biogenesis negative controller. It decides how much Father, or phosphatidic destructive, is present and how the lipids mix in the first parts of autophagosomes [35]. Expanded degrees of essential autophagy have been made sense of in *Caenorhabditis elegans*. Autophagy is set off by heat tension and force shock reaction record factor (HSF-1), which upgrade the worm's proteostasis and perseverance. Histone H3R17 dimethylation (histone H3 methylated at arginine 17) is a key epigenetic marker of autophagy that starts when cells are starving. It is followed by histone (coactivator-related) arginine methyltransferase, or CARM1. Moreover, it has been found that epigenetic rules change autophagy by histone (coactivator-related) arg [36].

Also, studies have shown that the vitamin D receptor may have an impact on autophagy in both solid mammary organs and luminal breast cancer cells. This exploration raises the possibility of a helpful connection between vitamin D levels and breast malignant growth risk. Various endogenous and outer factors might actually affect autophagy. Some of these factors are record factors, changes in the amount or collection of different biochemicals in the cytoplasm, damaged organelles, new drugs, and contamination. Accordingly, the parts behind autophagy may change [37].

There are different names for autophagy, such as macroautophagy, microautophagy, and CMA, depending on the tool used to move materials inside cells into the lysosome for destruction and the subatomic plans that target substrates in the lysosomes. Surprisingly, the movement of parts inside cells into the lysosome to be destroyed is thought to be macroautophagy, but the transport is thought to be microautophagy and CMA [38]. Albeit these pathways all go through debasement by means of the lysosome, their basic cycles contrast marginally from each other [39]. Most types of specific autophagy destroy specific targets, such as lipids, microorganisms, pexophagy, glycophagy, aggrephagy, and trama center phagy. Mitophagy targets mitochondria, pexophagy targets peroxisomes, and glycophagy targets protein totals [40]. A group of serine/threonine kinases make up the eIF2 kinase pathway. These kinases are limited during development and control translational capture that happens when cells are stressed. Inside this system, autophagy is an original activity that has developed to be rationed.

### 3.1 Macroautophagy

Macrautophagy happens when a piece of the cytoplasm incorporating a cell organelle is discarded to account for the autophagosome. The autophagosome connects with the lysosome or late endosomal multivesicular bodies (MVBs) to slow down the parts that are inside it. Autophagosomal protein Atg8 was first thought to be microtubule-related protein 1A/1B-light chain 3 (LC3) in people. It was quickly identified and studied [41]. There are two sorts of macroautophagy processes: cargo express and vague [42]. At the point when a yeast changes from non-fermentable to fermentable carbon sources, such as glucose, the course of mitophagy can be noticed. This is because of the extra mitochondria going through the mitophagy interaction following the change. Uth1p, a protein having a place with the SUN family, was quick to be distinguished as the reason for mitophagy in yeast. At the point when yeast is ravenous, this protein in the external mitochondrial film permits the yeast to dispose of excess mitochondria [43]. There are four types of mitophagy receptors: Func1 and BNIP3, BNIP3L/NIX, and SQSTM1/p62. They depend on hypoxia, erythrocyte growth, and damage-triggered mitophagy, in that order [44]. A receptor for specific autophagy is the protein Atg32, tracked down in the external layer of mitochondria [45].

Yet, among mammalian species, this receptor isn't moderated. In *Saccharomyces cerevisiae* and Pichia pastoris, pexophagy starts when the larvae's food source changes from oleic acid or methanol to glucose or nitrogen deficiency [46]. This is caused by the Atg36 and PpAtg30 receptors. *Saccharomyces cerevisiae* also experiences the enlistment of pexophagy. Besides, non-specific macroautophagy actuated by hunger has been illustrated, regardless of the way that autophagy requires mitochondrial phospholipids. Utilizing yeast cells, the instrument expected for particular autophagy has been totally examined. Based on these studies, it was found that vacuolar hydrolases are precisely moved into the vacuole of developing yeast cells using a pathway called CVT. A great thing about the CVT vesicles during autophagy in mammals is that the outer layers have a lot of shape. These layers are also known as phagophores, or partition layers [47].

### 3.2 Microautophagy

It is the job of lysosomal proteases to break down the contents of vesicles that were made by the lysosome by engulfing and creating space for small bits of cytoplasm. Microautophagy is the most common way that multi-vesicular bodies (MVBs) are made. MVBs are responsible for transporting solvent proteins to late endosomes. An important part of this cycle is the way that heat shock-related protein 70 (HSC70) and endosomal sorting complexes needed for transport I and III interact with each other electrically. Subsequently, components of both the endocytic and autophagic processes are incorporated into microautophagy [48].

### 3.3 CMA

**Fig. 3:**
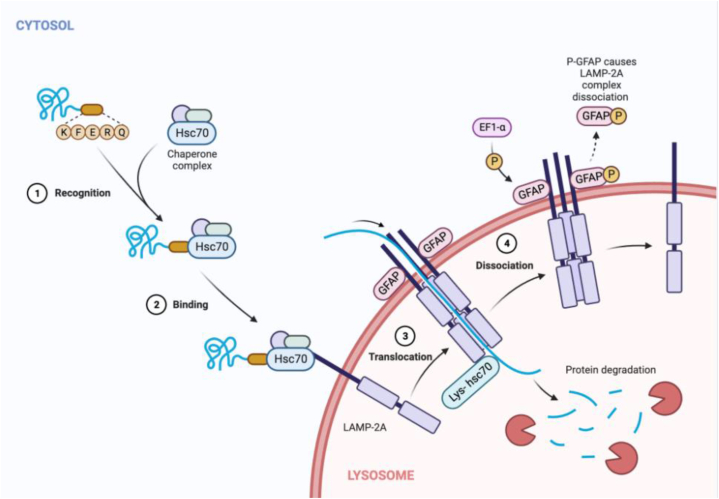
Chaperone-mediated autophagy (CMA) (Created with BioRender.com).

Just proteins that incorporate a C-terminal pentapeptide KFERQ theme can go through the CMA interaction; the HSC70 [49] Cochaperone is responsible for finding cytosolic proteins that have this grouping and transporting them to the lysosome [50]. It has been seen that chaperones attached to the substrate are moved to the surface of the lysosome, where they join forces with Light 2A in pure form [51]. It has been found that for Light 2A to move the substrate, it needs to first make a multiprotein complex. The improvement of the Light 2A complex is a remarkable cycle that happens when the substrate joins with the receptor. The spread-out substrate protein is hence transported into the lysosome by Light 2A (escort-mediated), where it will be obliterated. After this step, Light 2A is destroyed, and its monomers are ruined in lipid microdomains. At the level of the lysosomal movie, the speed of CMA is immovably coordinated by the levels of Light 2A. In the mammalian form of the viral watchman system, a cell-autonomous autophagy part has been found. This tool helps cells stay alive by letting viral proteins take part in autophagic clearance through the cell p62 adaptor. Viruses that use positive-strand RNA, like the flu and porcoraviruses, speed up the formation of autophagic films but stop them from fully developing [52]. So, looking into the links between autophagy and adenoviruses could help create oncolytic virotherapies based on adenoviruses [53]. Fig. 3 gives a visual portrayal of the CMA method.

## 4 Autophagy in drug delivery vectors

In order to preserve cellular homeostasis, autophagy is a normal cellular process that includes the breakdown and recycling of cellular components. It is essential for several physiological functions, including growth, immunity, and stress response. In order to increase the efficacy and specificity of medicinal treatments, researchers have recently begun to investigate the potential of autophagy in drug delivery systems [54]. Here is an example of how autophagy is used in drug delivery vectors.

● **Autophagy as a method for the delivery of drugs:** One strategy includes the development of drug delivery vectors (such as nanoparticles or liposomes) that make use of the autophagy process in order to accomplish targeted drug delivery. These vectors may be programmed to be recognized and internalized by cells by means of certain receptors, which will ultimately result in their encapsulation inside autophagosomes.
● **Modulation of autophagy for the control of drug release:** Researchers are able to alter the autophagy process in order to control drug release by focusing on certain molecular regulators of autophagy. For example, some stimuli or external triggers may activate autophagy and enhance drug release from the delivery vectors at the target site of action. This occurs when the drug is transported to the site of action.
● **Autophagy for intracellular drug delivery:** Autophagy may also be used to increase the intracellular transport of medications, particularly in difficult-to-reach cellular compartments such as lysosomes. This is a particularly useful use of autophagy. The effectiveness of drug delivery may be improved by using autophagic mechanisms to help transport drug-loaded vectors to the appropriate compartments of the cell.
● **Autophagy as a treatment for drug-resistant cells -** Autophagy has, in certain instances, been associated with drug resistance. Researchers are investigating methods to use this phenomenon to their advantage by co-delivering autophagy inhibitors with traditional medications. This strategy is called “co-delivery.” By inhibiting autophagy in drug-resistant cells and so rendering those cells more receptive to the curative effects of the medications, this strategy hopes to overcome the problem of drug resistance.
● **Autophagy-mediated immunomodulation:** There are some drug delivery vectors that may set off autophagy in immune cells. This can then lead to the presentation of antigens and the start of immune responses. The development of innovative immunotherapies might benefit from using this technique.
● **Autophagy and personalized medicine:** An understanding of the autophagic response of individual patients may be helpful in the development of personalized medication delivery systems that are adapted to the patients' particular requirements and circumstances.

It is vital to keep in mind that the area of autophagy-based medication delivery is still in its early phases and that a great deal of work needs to be done before these methods may be used in clinical settings. Researchers are now working hard to address some of the most important issues, including the complexities of autophagy control, the possibility of deleterious consequences, and the need for precise targeting. There are many different therapeutic approaches, and as research continues to develop, autophagy-based drug delivery vectors may provide novel and cutting-edge strategies to increase the efficacy and safety of these therapies.

## 5 Autophagy modulating drugs

According to the research of [55,56], autophagy is often linked to tumour survival and chemoresistance [57]. found that inhibiting autophagy might make chemoresistant cells more sensitive to the effects of chemotherapeutics, hence increasing the likelihood of tumour apoptosis. For example, it might be able to protect tamoxifen blocking in HER-positive breast cancer cells by decreasing Atg5, Atg7, or beclin1 in a way that is passed down through genes. When mixed with 3-Mama, an autophagy inhibitor, the HER2-explicit monoclonal immune response trastuzumab (Tmab) makes a bigger difference in fighting cancer. Cancer cells in the ovaries that are missing atg5 can go through apoptosis because they normally have increased autophagy and can't be killed by cisplatin [58].

The FDA presently perceives the main medications that slow down autophagy as protected and viable for use in clinical settings: the antimalarial drug chloroquine (CQ) and its subsidiary, hydroxychloroquine (HCQ). Concentrates [59] show that HCQ is a lysosomotropic drug that can stop lysosomal fermentation and, by extension, the autophagic stream. It is imperative that the action of CQ be fundamentally stifled in low pH conditions; this could represent the restricted impact of CQ in vivo since cancer microenvironments are generally acidic because of raised glycolytic rates and tissue harm [60]. The first stage of the study, which included patients with advanced non-small cell lung cell breakdown, showed that HCQ increased the viability of erlotinib (EGFR) [61]. It has been demonstrated that HCQ builds the action of mTOR inhibitors; for example, everolimus in cell lines comes from renal disease. This, thusly, brings down the amount of oxygen consumed by mitochondria and raises the chance of cell self-destruction by forestalling S6 phosphorylation. It was thought that the combination of tamoxifen (Cap) and hydroxychloroquine (HCQ) worked better than either drug alone at increasing the response to estrogen therapy in breast cancer cell lines that had estrogen receptors that were positive (trama center +) [62].

On the other hand, the death of tumour cells may also be caused by an accelerated form of autophagy induction brought on by cytotoxic chemical therapy or by direct autophagy induction. For instance, temozolomide is an alkylating drug that has pro-autophagy action. When temozolomide was mixed with dasatinib, a tyrosine kinase inhibitor, the two drugs worked together to kill glioblastoma cells. Inhibitors of histone deacetylase, often known as HDACs [63], as well as proteasome inhibitors (PI), which may possibly have a role in inducing autophagy: In prostate cancer cells, the PI Bortezomib causes a rise in the early production of autophagosomes and LC3-II expression. Bortezomib is used in the treatment of hematological malignancies [64]. Two well-known autophagy inducers, everolimus and temsirolimus, were seen as helpful in stage III preliminary studies for treating renal cell carcinomas that are getting worse. Everolimus has been endorsed by the FDA to treat progressive bosom disease, pancreatic neuroendocrine cancers, and renal cell carcinoma as an antiangiogenic drug. In spite of this, clinical preliminaries have shown huge obstruction rates to anticancer therapies that just depend on mTOR hindrance [65].

It can be seen that HCQ and mTOR inhibitors have been tested together in a number of stage I and stage II clinical trials as a possible way to treat advanced solid tumours, multiple myeloma, and kidney disease [66]. Patients with advanced colorectal cancer are being used in a preliminary clinical study (NCT01206530) that looks at the link between autophagy and the metabolic course of disease by combining HCQ with chemotherapy. They will be combined in a new study (NCT02042989) to see what happens to autophagy in people with advanced p53 freak tumours when proteasomes and HDAC inhibitors are used together. MLN9708 is a proteasome inhibitor, and vorinostat is a wide-reaching HDAC inhibitor. HDAC family members and other epigenetic regulators manage the activation of autophagy in a number of ways, such as by changing the quality of autophagic device focus expression. After HDAC inhibitor treatment, autophagy development fundamentally increases, thus diminishing the anticancer effect of HDAC inhibitors [[67], [68]]. Studies have shown that proteasome inhibitors can also boost autophagy, which is thought to make it easier for malignant growth cells to handle stress. At this point, everything is set up for the first phase and clinical studies of combined treatments that stop autophagy near HDAC or proteasome ability [69]. The increasing number of studies that are now being conducted is evidence of the importance that modulating autophagy may have in the development of combinatorial therapies that can overcome the resistance that is already present in certain cancer treatments. Recent studies show that autophagy causes big changes in metabolism and chemoresistance. It also has immunomodulatory properties that could be used to improve immunotherapy for diseases. This finding was made doable by the fact that autophagy causes extreme metabolic issues [70].

## 6 Nanostructures for delivery systems of autophagy modulators

The utilization of medications that are presently on the market that intervene in autophagy in clinical settings is hampered by a number of obstacles. Autophagy modulators aren't always bioavailable for a number of reasons, such as not working as well in acidic environments, not dissolving well in water, or not going where they're supposed to go in addition, autophagy modulators frequently display poor solubility. Recent years have seen the development of therapeutic solutions based on nanotechnology. These solutions are collectively referred to as nanomedicine [71]. A few nanoparticles (NPs) have been created that can act as imaging tools, notwithstanding medicine transporters. Certain physicochemical qualities, including charge, shape, surface improvement, and a high surface-to-volume proportion, are available in NPs. This might make them particularly attractive for stacking and conveying little mixtures to specific destinations [72].

By means of an enhanced permeability and retention effect (EPR), nanomedicine possesses the potential to mediate the preferential accumulation of systemically administered chemotherapy at tumour locations. In this case, the tumour microenvironment—which is typified by a restricted lymphatic drainage system and a leaky vascular system—helps to improve the specificity of the therapeutic approach when applied in in vivo settings [73].

Broad exploration of malignant growth nanomedicine has brought about the creation of nanostructures that might convey chemotherapeutic drugs to explicit places and defeat natural hindrances, all while limiting unfriendly consequences for sound tissues. It is possible to change the outer layer of NPs synthetically to add useful parts like nucleic acids and targeting ligands that can help them get to malignant growth areas more efficiently and make chemotherapy work better. There are a few techniques for finishing this [74]. In general, nanomaterials have been investigated for their potential as powerful modulators of autophagy through a number of different pathways, and they are also currently being examined as potential novel therapeutic weapons against cancer.

Cancer is still incurable in this day and age, despite being recognized as one of the primary causes of death among both younger and older people. This is due, in part, to the fact that our knowledge of its unique processes is limited and also to the fact that the activation of several signaling pathways inside cancer cells makes the treatment of cancer more difficult. It is interesting to note that nanomedicine has great potential for the treatment of cancer. This is due to the fact that cancer cells are more sensitive to certain NPs than normal cells are, which makes NPs a desirable choice for passive tumour targeting [75]. As was already discussed, more recent research has suggested that autophagy plays a significant role in the regulation of the stage at which tumours are found as well as neurodegenerative illnesses. In spite of the fact that a significant amount of research has previously been conducted on the subject of neurodegenerative illnesses, there has been a notable surge in research in this area lately [76]. In the field of drug delivery, some of the most significant obstacles are drug non-selective biodistribution, hydrophilicity, and cell absorption. It would suggest that encapsulating medications inside nanocarriers might be an effective strategy for overcoming these challenges [77]. Since nanoparticles (NPs) behave in the body as if they were foreign biomolecules and are about the same size as viruses and certain tiny bacteria, they have the potential to cause cells to increase their levels of autophagy [78]. The way that the size of NPs and transporters influences the organic activities they have is truly outstanding [79,80]. In outline, the fuse of autophagy modulators into nano-transporters is prescribed as a possibly successful way to tackle this issue, examples for nanoparticles participating in autophagy regulation are highlighted in Table 1.

**Table 1:** Nanoparticles in autophagy regulation.

Nanostructure	Applied agents in nanoparticle synthesis	Drug/gene delivery	Zeta potential (mV) Particle size (nm)	In vitro/in vivo	Cell line/animal model	Remarks	Refs
Polymeric nanoparticles	Selenium Hydroxyapatite	–	50–100 nm	In vitro In vivo	MNNG/HOS cells Orthotropic xenograft mouse model	Selenium plays a part in chemotherapy. Bone repair is provided by hydroxyapatite. Raising the amount of ROS JNK signaling being triggered and Akt/mTOR signaling being inhibited Causing cancer cells to undergo autophagy and apoptosis	[81]
Polymeric nanoparticles	Selenium	Laminarin Chloroquine	60 nm	In vitro	HepG2 cells	causing cancer cells to undergo both autophagy and apoptosis Up regulating p62 and LC3-II in the induction of autophagy Using chloroquine to block autophagy causes cell death	[82]
Polymeric nanoparticles	Selenium	–	30 nm	In vitro	HCT116 cells	Beclin-1 up regulation Activating autophagy in cancer cells to accelerate their demise	[83]
Polymeric nanoparticles (PLGA and succinate)	–	Doxorubicin Chloroquine	–	In vitro	A549 cells	doxorubicin's protection via autophagy inhibition and improved nuclear translocation of this chemotherapy drug	[84]
Polystyrene nanoparticles	Amino groups	–	+37 to − 56.7 mV	In vitro	OVCAR3 cells	Autophagy inhibition increases anti-tumour action that exhibits time- and concentration-dependent behaviour.	[85]
Polymeric nanoparticles	PEI PLGA	Paclitaxel	+21.7 mV 80 nm	In vitro	U251 cells	Preventing the growth and invasion of cancerous cells LC3-II and autophagosome accumulation are important in initiating autophagy.	[86]
Polymeric nanoparticles	–	HGFK1 Sorafenib	+6.68 mV 106.67 nm	In vitro In vivo	786-O, and ACHN cells Xenograft mouse model	inhibiting the development of tumours enhancing the survival of mice demonstrating the combinatorial effects of sorafenib reducing sorafenib-mediated autophagy by HGFK1 administration	[87]
Polymeric nanoparticles	PLGA	Curcumin GANT61	213.3 mV 190–400 nm	In vitro	MCF-7 cells	reducing the ability of cancer stem cells to self-renew lowering the survival of cancer cells by inducing both autophagy and apoptosis	[88]
Selenium nanoparticles	–	–	27.5 nm	In vitro	MCF-7 cells	Selenium nanoparticles and radiation have a synergistic effect by inducing autophagy and raising ROS levels.	[89]
Lipid nanoparticles	–	–	+29.8 mV Up to 136 nm	In vitro	Hela cells	Bcl-2 expression is down regulated during endoplasmic stress to initiate autophagy.	[90]
Au–Ag nanoparticles	Polydopamine	–	200 nm	In vitro In vivo	T24 cells Xenograft model	Delivering photo-thermal treatment and raising ROS concentrations Activating the ERK and Akt signaling pathway promoting both apoptosis and autophagy	[91]
Au nanoparticles	Valine	–	−29 mV 20 nm	In vitro In vivo	MDA-mB-231 cells Mouse model	Increasing ROS concentrations Activating autophagy Applying cytotoxicity to cancerous cells	[92]
Au nanoparticles	–	Quercetin	−19.1 mV 106.7 nm	In vitro In vivo	U87 cells Nude mice	decreasing cell viability in a way depending on concentration and time suppressing the expression of PI3K/Akt and mTOR Up regulation of ERK and LC3-II triggering autophagy	[93]
Super-paramagnetic iron oxide nanoparticles	–	AGO2 MiRNA-3765B	−20 mV 70 nm	In vitro In vivo	MCF7 and MDA-MB-453 cells Xenograft nude mice	demonstrating anti-tumour action and increasing cisplatin's cytotoxicity against cancer cells suppression of autophagy lowering the expressions of ATG4C and Beclin-1	[94]
Zinc oxide nanoparticles	–	–	20 nm	In vitro	SKOV3 cells	Cancer cell viability declining p53 and LC3 up regulation Encouragement of autophagy	[95]
Zinc oxide nanoparticles	–	–	−5.01 mV 172 nm	In vitro In vivo	MCF-7 cells Animal models of 4 T1 tumour cells	Increasing ROS concentrations Increasing ATG5 Activating autophagy	[96]
Hollow mesoporous silica nanoparticles	–	Hydroxychloroquine	+41.15 to − 26.50 mV 48.8 nm	In vitro In vivo	HCT116 cells Xenograft model	Growing hydroxy-chloroquine intracellular accumulation preventing autophagy as a means of promoting survival Increasing radiation's cytotoxicity for cancer treatment	[97]
Silica nanoparticles	–	–	86 nm	In vitro	HCT116 cells	Colon cancer treatment via influencing the endoplasmic reticulum to induce autophagy raising the LC3-II levels	[98]
Mesoporous silica nanoparticles	Poly-dopamine	Chloroquine Glucose consumer glucose oxidase	Up to 235 nm	In vitro In vivo	HepG2 cells Tumour bearing mice	The malnutrition that GOx causes in cancer cells Providing photo-thermal treatment Increasing the potential for autophagy inhibition to decrease cancer by fasting and photo-thermal treatment	[99]
Cuprous oxide nanoparticles	–	–	–	In vitro In vivo	J82, T24, 5637, UMUC-3 cells Xenografts	Causing apoptosis and cell cycle arrest; decreasing the survival of cancer cells in a way depending on time and concentration to increase apoptotic cell death, ERK signaling up regulation and autophagy activation are important.	[100]
TiO2 nanoparticles	–	5-Fluorouracil	20–30 nm	In vitro	AGS cells	Raising ROS levels causing lysosomal function disruptions inhibiting autophagy encouraging chemotherapy's cytotoxicity bringing about apoptosis	[101]
Magnetic iron nanoparticles	PEI	–	25.1 mV 26.3 nm	In vitro	HeLa cells	Increasing ROS concentrations ATG7 up regulation and activation of the Akt/mTOR axis triggering autophagy	[102]
Polymeric nanoparticles	Selenium	Laminarin Chloroquine	60 nm	In vitro	HepG2 cells	causing cancer cells to undergo both autophagy and apoptosis Up regulating p62 and LC3-II in the induction of autophagy Using chloroquine to block autophagy causes cell death	[103]
Polymeric nanoparticles	Selenium	–	30 nm	In vitro	HCT116 cells	Beclin-1 up regulation Activating autophagy in cancer cells to accelerate their demise	[104]
Polymeric nanoparticles (PLGA and succinate)	–	Doxorubicin Chloroquine	–	In vitro	A549 cells	doxorubicin's protection via autophagy inhibition and improved nuclear translocation of this chemotherapy drug	[105]
Polystyrene nanoparticles	Amino groups	–	+37 to − 56.7 mV	In vitro	OVCAR3 cells	Autophagy inhibition increases anti-tumour action that exhibits time- and concentration-dependent behaviour.	[106]
Polymeric nanoparticles	PEI PLGA	Paclitaxel	+21.7 mV 80 nm	In vitro	U251 cells	Preventing the growth and invasion of cancerous cells LC3-II and autophagosome accumulation are important in initiating autophagy.	[107]
Polymeric nanoparticles	–	HGFK1 Sorafenib	+6.68 mV 106.67 nm	In vitro In vivo	786-O, and ACHN cells Xenograft mouse model	inhibiting the development of tumours enhancing the survival of mice demonstrating the combinatorial effects of sorafenib reducing sorafenib-mediated autophagy by HGFK1 administration	[108]
Polymeric nanoparticles	PLGA	Curcumin GANT61	213.3 mV 190–400 nm	In vitro	MCF-7 cells	reducing the ability of cancer stem cells to self-renew lowering the survival of cancer cells by inducing both autophagy and apoptosis	[109]
Selenium nanoparticles	–	–	27.5 nm	In vitro	MCF-7 cells	Selenium nanoparticles and radiation have a synergistic effect by inducing autophagy and raising ROS levels.	[110]
Lipid nanoparticles	–	–	+29.8 mV Up to 136 nm	In vitro	Hela cells	Bcl-2 expression is down regulated during endoplasmic stress to initiate autophagy.	[111]
Au–Ag nanoparticles	Polydopamine	–	200 nm	In vitro In vivo	T24 cells Xenograft model	Delivering photo-thermal treatment and raising ROS concentrations Activating the ERK and Akt signaling pathway promoting both apoptosis and autophagy	[112]
Au nanoparticles	Valine	–	−29 mV 20 nm	In vitro In vivo	MDA-mB-231 cells Mouse model	Increasing ROS concentrations Activating autophagy Applying cytotoxicity to cancerous cells	[113]
Au nanoparticles	–	Quercetin	−19.1 mV 106.7 nm	In vitro In vivo	U87 cells Nude mice	decreasing cell viability in a way depending on concentration and time suppressing the expression of PI3K/Akt and mTOR Up regulation of ERK and LC3-II triggering autophagy	[114]
Super-paramagnetic iron oxide nanoparticles	–	AGO2 MiRNA-3765B	−20 mV 70 nm	In vitro In vivo	MCF7 and MDA-MB-453 cells Xenograft nude mice	demonstrating anti-tumour action and increasing cisplatin's cytotoxicity against cancer cells suppression of autophagy lowering the expressions of ATG4C and Beclin-1	[115]
Zinc oxide nanoparticles	–	–	20 nm	In vitro	SKOV3 cells	Cancer cell viability declining p53 and LC3 up regulation Encouragement of autophagy	[116]
Zinc oxide nanoparticles	–	–	−5.01 mV 172 nm	In vitro In vivo	MCF-7 cells Animal models of 4 T1 tumour cells	Increasing ROS concentrations Increasing ATG5 Activating autophagy	[117]
Hollow mesoporous silica nanoparticles	–	Hydroxychloroquine	+41.15 to − 26.50 mV 48.8 nm	In vitro In vivo	HCT116 cells Xenograft model	Growing hydroxy-chloroquine intracellular accumulation preventing autophagy as a means of promoting survival Increasing radiation's cytotoxicity for cancer treatment	[118]
Silica nanoparticles	–	–	86 nm	In vitro	HCT116 cells	Colon cancer treatment via influencing the endoplasmic reticulum to induce autophagy raising the LC3-II levels	[119]
Mesoporous silica nanoparticles	Poly-dopamine	Chloroquine Glucose consumer glucose oxidase	Up to 235 nm	In vitro In vivo	HepG2 cells Tumour bearing mice	The malnutrition that GOx causes in cancer cells Providing photo-thermal treatment Increasing the potential for autophagy inhibition to decrease cancer by fasting and photo-thermal treatment	[120]
Cuprous oxide nanoparticles	–	–	–	In vitro In vivo	J82, T24, 5637, UMUC-3 cells Xenografts	Causing apoptosis and cell cycle arrest; decreasing the survival of cancer cells in a way depending on time and concentration To increase apoptotic cell death, ERK signaling up regulation and autophagy activation are important.	[121]
TiO2 nanoparticles	–	5-Fluorouracil	20–30 nm	In vitro	AGS cells	Raising ROS levels causing lysosomal function disruptions inhibiting autophagy encouraging chemotherapy's cytotoxicity bringing about apoptosis	[122]

**Fig. 4:**
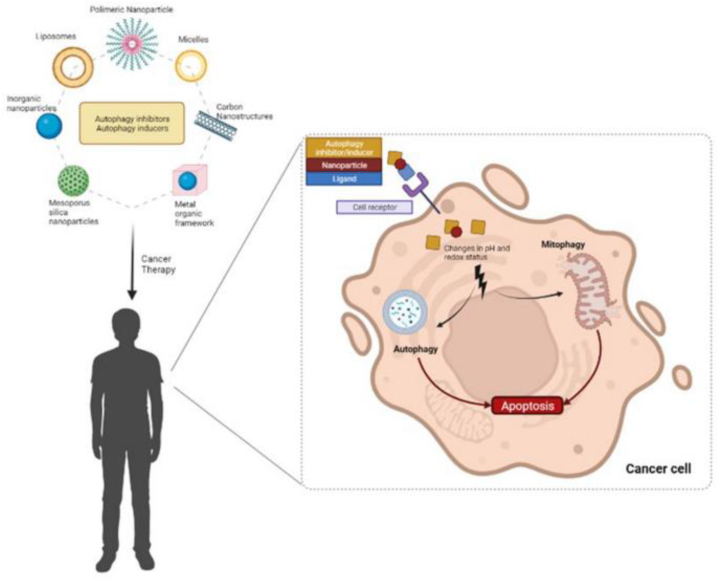
Several nanocarriers are used to deliver autophagy modulators [125].

When utilized as a transporter for autophagy modulators, nanoparticles give various advantages, including high conveyance viability, negligible systemic toxicity, and the evasion of medication opposition. There is room for using more traditional transporters, such as liposomes, micelles, and polymeric nanoparticles [123], to treat autophagy (Fig. 4). However, some experts know that using metal nanoparticles like iron oxide, silver, and gold alone, with autophagy drug silencers, or in combination with an outside inducer (like laser openness), may cause too much autophagy inhibition. A small amount of research has also been done on the effects of different nanomaterials on disease treatment, such as molybdenum disulfide (2D nanosheets), lipid calcium phosphate nanoparticles, and nanogels [124].

Human-made structures with no less than one aspect falling somewhere in the range of one and one hundred nanometers are known as nanostructures [126]. Besides, their physical and compound properties vary from those of recoil materials [127]. Nanomaterials can have one, two, or three things going for them. These include nanosheets (graphene oxide), nanofibers [128], carbon-based NPs [129], quantum dabs, polymeric, clay, or metallic NPs [130], liposomes, micelles, and different designs. As indicated by Wilhelm et al., dynamic prescription conveyance is essentially more successful when pole-formed inorganic nanocarriers with unbiased zeta potential and a molecule size of under 100 nm are focused on strong growths instead of organic nanocarriers with one or the other positive or negative zeta potential. As recently referenced, autophagy modulators might be reconstituted into nanocarriers to improve their clinical interpretation potential [131]. This guides in beating deterrents, including lacking solvency, confined solidity, and customized bioavailability.

Made a chemotherapeutic medicine utilizing 3-Mama-containing metal-organic framework (MOF) nanoparticles [132]. These three proteins work together to stop autophagy by lowering the class III PI3K (Vps34)/Beclin-1 complex. This stops the formation of autophagosomes. Examination showed that high convergences of the 3-Mama epitome (19.798 wt%) essentially repress the development of autophagosomes compared with free 3-Mama and meaningfully affect HeLa cells over the long run. This is a critical perception, as it proposes that high 3-Mama exemplification levels unequivocally hinder autophagosome creation. A different report said that [133] made zeolitic imidazole nanoparticles (ZIF-8) that target cancer and are typical of CQ. polyethylene glycol-folate (FA-Stake) covering was applied to them in an effort to help the amount of medicine that the NP retained. These discoveries showed that autophagy restraint altogether expanded the death pace of disease cells.

### 6.1 Liposomes

Liposomes are widely regarded as the very first medication nanocarrier to be made available for commercial use in the treatment of cancer, which occurred in 1995. On the other hand, it is widely used as a medication for the treatment of a wide variety of illnesses [134]. Because of their sensitivity to changes in pH and temperature, liposomes are an excellent choice for tumour detection and targeting, particularly when it comes to photothermal treatment**.** In terms of the components that make up liposomes, it has biocompatibility, reduces the risk of medication biodegradation, increases drug solubility, and possesses target-specificity [135]. In view of these qualities, liposomes are believed to be a valuable drug conveyance method [136]. To fix malignant growth [137], fostered a pH-delicate and plasma stable liposome with rapamycin going about as an autophagy inhibitor and monomethyl itaconate as a lipid establishment. When compared to rapamycin on its own, the researchers' findings showed that liposomes containing rapamycin were superior in terms of their effectiveness, level of cytotoxicity, and ability to limit cell growth [138]. PEGylated liposomes containing metformin and epirubicin were tested to see how effective they were in combating CD^133+^ cancer stem-like cells. According to the results of their research, the cytotoxicity of liposomes containing metformin and epirubicin is much greater than that of metformin and epirubicin on their own. Liposomes containing simvastatin were created by Ref. [139] so that they could investigate the drug's potential to suppress the development of tumours. They employed B16.F10 melanoma tumours and demonstrated that liposomes containing simvastatin significantly suppress the development of tumours by blocking the intratumor production of HIF-1α. This was done using the experimental model.

In addition to the function that liposomes play in passive targeting, it is possible for them to act as active targeting agents via the binding that they do with ligands like antibodies. For example [140], rapamycin was delivered to TNF-stimulated cells using decorated E-selectin antibody liposomes that were produced earlier. They postulated that the method would reduce the negative effects that rapamycin had on other cell types.

### 6.2 Micelles

Micelles are characterized as different sorts of nanocarriers, and how much energy is expected for their creation could fluctuate incredibly. It's important to use co-polymers and surfactants that are amphiphilic and have different hydrophilic-lipophilic balances (HLB) when making micelles [141]. In the event that the micelle is designed as water in oil or oil in water, the hydrophobic and hydrophilic bits will be tracked down on the shell or center separately. The method of making micelles is simple, and the fact that their core and shell may switch places gives them the ability to perform several functions and react to external stimuli. Micelles have been shown to have these characteristics. Because micelles are better at dissolving, staying stable, and spreading throughout the body than most pharmaceuticals, they are a great choice for encasing autophagy inhibitors. This is especially noteworthy given that micelles demonstrate better solubility than conventional medications [142]. cancer treatment using micelles containing rapamycin that were previously produced. By regulating rapamycin-micelles in HCT 11b and HeLa cells, they exhibited that the malicious impacts of the medication were more noteworthy than those of rapamycin alone on the suitability of cells. Also [143], pH-delicate micelles containing rapamycin were used to treat glioblastoma multiforme (U87 MG-cell line), and this had a stronger effect on cell death than rapamycin alone [144]. Film dialysis was used to make a micelle containing simvastatin, and its effects on MG-63 cells that look like human osteoblasts were studied. Based on their research, simvastatin has a supported delivery system that improves the growth and mineralization of osteoblast cells through the BMP-2 pathway. The scientists approved this. Subsequently, simvastatin-containing micelles might be considered a competitor for the treatment of breaks [145].

### 6.3 Polymeric nanoparticles

It is generally agreed that biodegradable polymer NPs represent the most significant nanoplatform for use in drug delivery applications [146]. In addition to their great entrapment effectiveness, the polymeric NPs have a high degree of both stability and the capacity to release the medication in a way that is within the control of the user [147]. Different biological effects, like biocompatibility, can be shown by liposomes and polymeric nanoparticles depending on what they are made of. On the other hand, a number of investigations have shown that liposomes have a limited range of applications due to their uncontrolled release, unstable storage conditions, and insufficient drug loading [148]. Polymer-hybrid lipid nanoparticles, or polymerosomes, benefit from the benefits of both liposomes and biodegradable polymers. Nonetheless, polymer-crossover lipid NPs or polymerosomes probably won't have the disservices of biodegradable polymers or liposomes. They have attractive pharmacokinetic properties, a serious level of biocompatibility, and empower the medication to be delivered in a controlled way. It was studied in Ref. [149] how adding rapamycin to a polymer-lipid nanoparticle (NP) affected mice with subcutaneous hemangioma and how the nanocarriers affected the mice. Their outcomes showed that hemangioma altogether diminished the survivability of creatures holding onto nanocarriers in mice when contrasted with rapamycin alone.

Notwithstanding polymer-lipid NPs, polymeric nanoparticles (NPs) may possibly be researched for the therapy of malignant growth. Pegylated poly(lactic corrosive) nanoparticles (PLGA NPs), which have a high cell take-up limit, can be used to move the drug dactolisib to a specific location. This makes these NPs a good choice for drug delivery. Moreover [150], blended and made a cutting-edge nanocarrier for topically conveying everolimus. Prior to stacking everolimus into the nanocarriers, they made a nanostructure known as methoxy-poly (ethylene-glycol)-hexyl-subbed poly (lactic corrosive), or mPEGhexPLA. It's essential to see that topical nanocarrier organization diminishes lymphocyte invasion while likewise meaningfully affecting the spleen's invulnerable reaction. In an alternate report, Ding et al. incorporated a thermoresponsive nanocomposite gel determined to set off autophagy to stop glioma development. They treated C6 cells with the nanocarriers and exhibited that paclitaxel is more successful against these cells than temozolomide. Moreover, they showed that, by enacting autophagy, the combinational treatment had higher cytotoxic impacts [151]. This was a huge disclosure. Moreover, they showed that neighborhood drug conveyance by means of the nanocomposite gel intensifies against glioma cancer exercises [152]. Gefitinib was integrated into carboxymethyl-beta-cyclodextrin nanoparticles connected with ox-like serum egg whites. The folate-decorated NPs were utilized to improve the organization of medication. In HeLa cells that have folate receptors, the nanocarriers help the caspase-3 protein connect and slow down ATP combination, both of which speed up the cell death process. The limit of the nanocarriers' ability to down-direct the LC3 protein represses autophagy [153]. Utilizing lipoplex and polyplex NPs, Tune et al. concentrated on the role of autophagy in the intracellular transport of irregular siRNA. It was shown that autophagy can continue without the mTOR pathway. A number of autophagy regulators can also significantly improve or decrease the effectiveness of siRNA knockdown, which changes how siRNA is distributed inside the cell. Therefore, autophagy modulators might be thought of as a likely method for accomplishing designated quality hushing. Zhang and partners delivered PLGA NPs in an alternate report to investigate the effect autophagy inhibitors have on the transportation of medications used to treat disease. As per the examination, autophagosomes are accountable for inundating NPs that have gotten away from the endolysosome and directing them into the lysosomes, where they could go through debasement. This makes sure that nanoparticles with autophagy inhibitors like 3-Mama and CQ can move along the intracellular drug delivery pathway. To expand their protection from chemotherapeutic treatment, disease cells effectively enact autophagy, a supportive endurance system [154]. For this explanation, one could argue that PLGA NPs containing autophagy inhibitors are useful for an expansion in their restorative benefits.

## 7 Co-delivery of chemotherapeutics/siRNA and autophagy inducers/inhibitors

So, fully understanding the stages of cancerous growth, changes, variations in signaling pathways, and related processes is important for creating an effective drug delivery system that might be able to treat disease. The possibility that ATP-cutthroat inhibitors of mTOR and even rapamycin could increment drug obstruction, a condition welcomed by mTOR transformations that can arise, is a puzzle. Be that as it may, it exactly happens. That being said, mTORC1 quality changes happen suddenly in an assortment of malignant growth types [155]. It has been shown that a combination treatment containing pazopanib, a tyrosine kinase inhibitor, and rapalog, an everolimus, is exceptionally effective in treating patients' malignancies [156]. It has been noticed that medications that disturb the survivin protein can make drugs like everolimus ineffective. In excess of ten FDA-supported clinical preliminaries, including the autophagy inhibitors CQ and HCQ, have now been finished or are right now in stages I and II for the therapy of different strong growths, including pancreatic disease, glioblastoma, and astrocytoma, prostate malignant growth, little and non-little cell cellular breakdown in the lungs, refractory or backslid numerous myelomas, bosom malignant growth, colorectal malignant growth, and others [157]. On the other hand, a lot of data from early tests suggests that stopping both mTOR and autophagy at the same time in growth cells can make them more harmful to other cells. It talks about a stage I/II clinical trial in which 38 people with clear cell renal carcinoma who had just finished a 1–3 VEGF-TKI protocol were given an oral mix of HCQ, which blocks autophagy, and everolimus, which blocks mTOR. A VEGF-TKI regiment had been utilized to treat every one of these people. The preliminary I results were used to recognize the best doses of HCQ (600 mg two times a day) and everolimus (10 mg every day), which were provided orally. While under 10 % of patients experienced grade 3–4 antagonistic occasions, most patients had the option to tolerate the medication at the suggested measurements, and most unfriendly occasions, including rash, sickness, exhaustion, paleness, and the runs, were grade 1–2. HCQ didn't demolish the toxicity of everolimus, and the joint treatment was very well tolerated, with a PFS rate of more than 40 % at 6.3 months. In any case, prompting transformations in the mTOR flagging pathway came about in a diminished PFS inside this regiment [158].

As expected, many studies have shown that combination therapies work. These include an autophagy inhibitor (LY294002), wortmannin, CQ, and minimal meddling RNAs along with a standard chemotherapy drug (like docetaxel, paclitaxel, doxorubicin (DOX), cisplatin, and 5-fluorouracil) to improve chemotherapy and get around drug resistance [159] in Area 5.

**Fig. 5:**
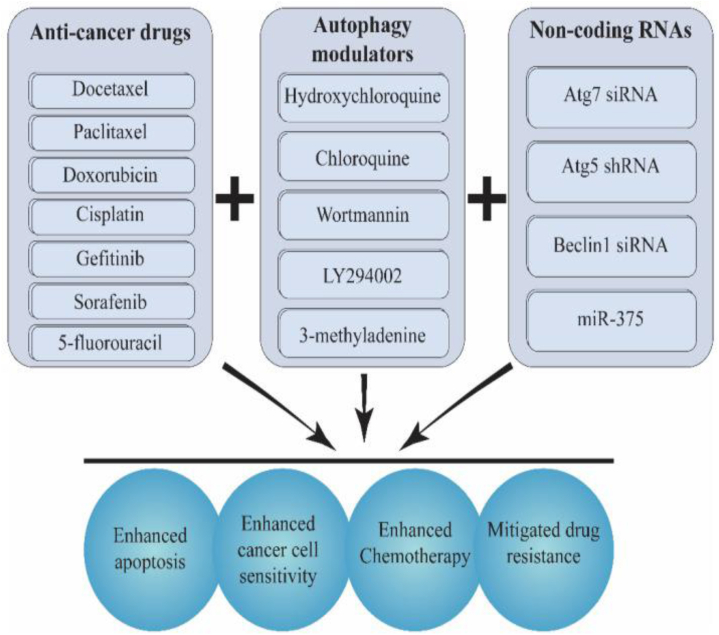
Chemotherapy is more effective when administered in conjunction with autophagy gene modulators or autophagy regulators [161].

Then again, long-haul docetaxel organization may emphatically direct autophagy, prompting chemo-obstruction and the growth's capacity to endure chemotherapy, potential strategies are demonstrated in Fig. 5. To do tests both in the lab and in living things [160], recently stacked siAtg7 and DTX into iRGD changed Pluronic P123-PEI co-polymer micelles. The iRGD peptide upgraded the drug's entrance and aggregation in creature tests. By effectively turning off Atg7 with siRNA and lowering LC3, treating with siAtg7 and DTX together increased the ability to heal. This brought about the down-controlled autophagy brought about by DTX in pancreatic malignant growth cells and the PANC-1 xenograft mouse model.

Also, to get around the hypoxic segments of growth's helplessness with the impacts of mTOR inhibitors, RNA obstruction or acetazolamide, an inhibitor of carbonic anhydrases, have been proposed as potential combos with mTOR inhibitors like rapamycin. This is finished to get around the hypoxic areas' protection from mTOR inhibitors' belongings. It's intriguing to take note that the acidic pH microenvironment of malignant growth cells is predictable with the idea of hypoxia inhumanity toward mTOR. The acidic microenvironment encompassing the disease directs which of the various upstream signal transduction pathways are trailed by flagging pathways [162]. The movement of mTORC1 in disease cells decreases because of acidic pH excitement, and the counterproliferative impact of mTOR inhibitors is, as of now, not compelling [163]. It has been recommended that alkalizing the malignant growth microenvironment with sodium bicarbonate will counterbalance this impact and increment the counterproliferative activity of mTOR inhibitors with regards to an acidic cancer microenvironment. Regardless, the ideal methodology is to foster a mixed treatment that incorporates an autophagy inducer system separate from mTOR. Still, a combination of everolimus and exemestane was suggested as a possible treatment for people with chemical ward breast cancer [164].

As recently referenced, MAPK enactment is an extra-flagging pathway that could start the autophagy interaction. Various examinations have analyzed the countergrowth opposition of MAPK and its phosphatase (MKPs), including protection from mechlorethamine, paclitaxel, DOX, and tamoxifen. Regardless of the way that causticity affects MAPK actuation, there are certain cases where this obstruction exists [165].

Aside from actuating autophagy, new exploration has exhibited that fasting upgrades the viability of cancer drugs managed in chemotherapy while at the same time alleviating the unfavorable impacts of the therapy and improving pressure tolerance [166]. It is nothing similar to what the American Malignant Growth Society proposes for disease patients with regards to unhealthy and protein admission. Remarkably, fasting builds the effectiveness of chemotherapy on dangerous cells while safeguarding sound cells from the possibly deadly impacts of the medication and advancing tissue recovery in solid cells. For instance, Groot et al. found that concise fasting stops the decay of platelets and RBC in patients with HER2-negative bosom disease and, somewhat additionally, stops DNA harm.

There are various occasions of blend treatments that utilize autophagy inhibitors notwithstanding those that contain autophagy inducers. HCQ is one of numerous pre-clinical autophagy inhibitors that has gotten FDA endorsement. It achieves this by repressing autophagosome obliteration and lysosomal fermentation, two fundamental stages in autophagy [167]. We talk about the utilization of HCQ in clinical preliminaries and autophagy inhibitors in preclinical exploration in this audit. These two sorts of investigations have both been done. It is vital to recall that even at high portions, HCQ just slightly restrains autophagy; moreover, its belongings are nullified in conditions with an acidic pH.

In pre-clinical stages, a mix of medicines, including autophagy inhibitors, has been examined. Seeing if or whether drug NPs enact the autophagy system is essential. A review led by Ref. [168] showed that the PEGylated PLGA micelle both initiates autophagy and might be killed inside autophagosomes. Utilizing the MCF-7 cell line, Gong et al. exhibited an effective mix treatment for the therapy of bosom disease. This treatment contained Atg7 siRNA crosslinked to docetaxel in a micellar formulation. In their study, they found that ensnarement inside the cancer site slowed down growth in mice and improved apoptosis and autophagy in disease cells. The way that the malignant growth cells were disposed of all the more effectively added to this to a limited extent. One more concentration on the concealment of Atgs is connected to plasmid DNA that communicates short clasp RNA, which is responsible for quieting Atg5 as a part of autophagy [169]. To make a mixed treatment strategy, shRNA targeting Atg5 and gefitinib were joined with chitosan nanoparticles (NPs) as a nanocarrier. The scientists found that in PCL and A549 cells, their discoveries demonstrated a higher bioavailability of nanocarriers, which corresponded with an ascent in apoptosis and a decrease in autophagy. Be that as it may, the blend treatment with nanocarriers was effective in bringing the cancer's volume down compared with the stripped mix therapy. Lipid-covered calcium carbonate nanoparticles (NPs) exemplifying autophagy inhibitor miR-375 and chemotherapeutic sorafenib (HCC) increase the cell passing of HepG2 cells in comparison with sorafenib. Besides, mixing treatments altogether decreases the cancer's volume. Prominently, the consolidated treatment might forestall autophagy welcomed by sorafenib-containing chemotherapy [170]. Moreover, LY294002 is an inhibitor of autophagy that targets PI3K. To cure disease, Saiyin et al. created a micelle of a polymeric hyperbranched polyacylhydrazone that had DOX and LY294002 in it. They had the option to show that in HN-6 and CAL27 cells, consolidated treatment advances apoptosis and represses autophagy. In fact, it's interesting that they showed that combining LY294002 into a single nanocarrier improves apoptosis and autophagy inhibition in cancer cells compared to giving LY294002 and nano DOX separately [171].

They continued with our examination of blend treatment, including HCQ, to survey the unparalleled autophagy inhibitor that has gotten FDA endorsement. Consolidated treatment with HCQ and paclitaxel exemplified into liposomes on melanoma cells brought about concealment of metastasis by means of down-guideline of MMP2 and 9 as well as decline of growth advancement, somewhat because of expanded maintenance in cancer site. Zhang et al. found that joined treatment with HCQ and Docetaxel typified into a PEGylated PLGA micelle brought about expanded cell mortality of MCF-7 cells notwithstanding a decline in growth volume in e SCID mice that had been infused with MCF-7 cells [[172], [173]].

To evaluate the combination treatment in human and clinical settings, further significant investigations will be examined in addition to the pre-clinical investigation. Patients with advanced pancreatic adenocarcinoma were given HCQ (600 mg/w) along with gemcitabine and nab-paclitaxel as part of a phase I clinical study (NCT01506973). The patients responded well to the treatment, and their data revealed that the combo drug may block autophagy. Subsequently, they showed in a phase II clinical trial that the percentage of patients with metastatic cancer who survived was not increased by combination treatment [174]. looked at how well patients with glioblastoma multiform responded to treatment that included both standard chemotherapy and CQ (150 mg/d). The overall survival rate was higher with combination treatment than with monotherapy, although the difference was not statistically significant [69]. It has been demonstrated that bortezomib and HCQ therapy together result in 45 % disease stability in myeloma patients. In a different experiment, a combination of temozolomide and HCQ was administered to melanoma patients. The study's findings demonstrated that patients were able to take the combo drug without experiencing any negative side effects [175]. inquired about clinical trials, including combination therapy. Based on what they found, we don't know if the combination treatment of HCQ (600 mg/d) with temozolomide, vorinostat, bortezomib, and temsirolimus starts autophagy at the right level or if this dose works better with chemotherapy. Additionally, they discovered that it's uncertain if this amount has a synergistic effect with chemotherapy.

The best methodology, as per concentrates by Ref. [176], was to utilize a nanocarrier that could all the while get across the twofold physiological boundaries of cancers. To empower the co-organization of chemotherapeutic medications, like DOX and autophagy inhibitors (3-Mama), the nanocarrier was planned with a framework metallopeptidase 2 (MMP2) degradable shell and a pH-set-off reversible expanding contracting center. This nanocarrier showed a huge level of viability in entering the growth's center in both in vitro and in vivo tests. Moreover, a synergistic anticancer impact was shown when DOX and 3-Mama were consolidated.

The potential of size-adjustable DSPE-PEG micelles containing wortmannin and DOX to inhibit autophagy and restrict tumour growth was investigated in a study conducted by Rao and colleagues. It was proven that autophagy was effectively stopped when LC3-II expression went down and p62 expression went up at the same time in B16F10 and 4T1 cell lines. Tiny particles have the ability to swiftly exit the circulation while still entering deep tissue [177]. Particles whose size increased and which became trapped in the extracellular matrix had an increase in retention time. This dual-drug nanoparticle demonstrated anti-tumour efficaciousness and suppressed autophagy in mouse models of breast cancer and melanoma.

In a different study [178], therapeutic nanohybrids were made by mixing gold nanoparticles (which block pores) and ROS (which release oxygen) with amino-functionalized mesoporous silica nanoparticles (MSNs), which have a high loading capacity for a chemotherapeutic drug to get rid of human non-small cell lung cancer (NSCLC). Gold nanoparticles and amino-functionalized MSNs were combined to create these nanohybrids. Chemotherapy and autophagy, which is caused by oxidative stress, worked well together thanks to the nanohybrids, creating a new way to treat cancer.

Utilizing three unmistakable multifunctional nanoplatforms [179], made a systemic co-conveyance methodology for rapamycin and celecoxib (CXB) all through their examination. This strategy was utilized to limit the development of bosom disease cells. These remedial nanocapsules were first planned with an oil supply to take into consideration the double stacking of rapamycin and CXB. Then, they were electrostatically covered with oppositely charged proteins (gelatin) and polysaccharides (chondroitin) that were connected to fluorescent quantum specks. The light trait of these nanostructures made it conceivable to monitor their assimilation inside malignant cells. Besides, it was shown that they fundamentally harmed bosom malignant growth cells.

In an alternate report, LY294002, an autophagy inhibitor, and docetaxel, a notable anticancer medication, were both stacked into PLGA nanoparticles. This study is planned to tackle the issues of hydrophobicity and low bioavailability that both docetaxel and LY294002 show. Because of the EPR effect [180], 155.3 nm PLGA (Docetaxel + LY294002) NPs were moved in the growth site of animals that were given a stomach disease xenograft model. It's possible that LY294002's blocking of the PI3K/AKT signaling pathway will stop metastasis by lowering the levels of MMP2, MMP9, and VEGF. This NP showed controlled discharge and a critical level of apoptosis in both in situ and xenograft mouse models of gastric cancer growth.

Cisplatin is an intense anticancer medication that has been generally used to treat a wide assortment of malignant growth types. In any case, cisplatin obstruction represents a serious test for the cancer therapy technique. The utilization of autophagy inhibitors, such as wortmannin and siRNAs, has all the earmarks of being a promising strategy for supporting cell responsiveness and beating drug obstruction, as per [181]. In a new report, wortmannin and cisplatin, two anticancer medications, were regulated all the while within PLGA-Stake NPs. The amount of H2AX foci, a marker for DNA twofold strand breaks, essentially diminished, demonstrating that wortmannin can hinder DNA fixation and restrain autophagy. This blend drug had the option to defeat cisplatin resistance and increase the viability of chemoradiotherapy in mouse models of PSOC and PROC malignant growth [182]. made a prodrug in an alternate report, fully intent on overcoming the cisplatin drug opposition. Using DSPE-Stake nanoparticles adjusted with DSPE-Stake and cRGD chains with a typical width of 55 nm, cisplatin (Pt IV) and Beclin1 siRNA were stacked for proficient medication organization and autophagy concealment in disease cells. The middle part of the molecule has cisplatin attached to a cationic peptide (KTGRKKRRQRRRG), which has been modified by bis(pyrene) and siRNA. Studies conducted in vitro and in vivo have shown the effective quieting of Beclin1 by this self-collected prodrug, which has a mean breadth of 54 nm. At the point when Beclin1 articulation and LC3-II levels were brought down, the cisplatin-safe growth in the xenograft mouse model showed expanded chemotherapy, diminished drug opposition, and forestalled disease movement. These impacts were additionally enhanced by an overall decrease in the mRNA levels for TFEB1 and ULK1. It very well might be shrewd to target disease cells utilizing a method that gains from the huge amount of energy needed by growth tissues. Due to their quick digestion, disease cells overproduce the proteins that tight spot egg whites, which makes it feasible for the cells to incorporate egg whites and use them as an amino acid inventory [183]. created folic corrosive-adjusted paclitaxel/CQ nanoparticles to target glioma cells and upgrade blood-cerebrum hindrance travel. CQ had the option, as far as possible, of autophagy by endosomal fermentation, raising LC3-II to LC3-I level, stopping over-articulation of SQSTM1/P62, and autophagosome combination with the lysosome. Scientists found that changed paclitaxel/CQ nanoparticles, which had an average size of 53 nm, were better at killing brain cells in T98G and LN229 mice than paclitaxel alone.3 cells, which are cells that line the innervation of the mind.

Relatively recently [184], created PEGylated nanoliposomes containing 5-fluorouracil as an anticancer drug and LY294002 as an autophagy inhibitor to treat esophageal squamous cell carcinoma (ESCC). This co-loaded cargo's outcomes showed that LY294002 was released more quickly than 5-fluorouracil. This means that autophagy is suppressed before the drug is released, making cancer cells more vulnerable to 5-fluorouracil therapy. These 150 nm-sized nanoliposomes downregulated the expression of the anti-apoptotic protein Bcl-2 on the EC 9706 esophageal cancer cell line while concurrently upregulating the expression of caspase-3 and PARP. The in vivo mouse model demonstrated both a delay in the advancement of tumour formation and a progressive release of carcinogenic byproducts into the surrounding acidic environment as compared to the control group. All things considered, combination treatments involving autophagy inducers and inhibitors require more study, and their clinical results differ significantly from pre-clinical studies. This is partially due to the fact that human cancer is a more complex disease than animal cancer models.

## 8 Challenges of designing a drug delivery system considering nanocarriers' role as autophagy modulators

As recently referenced, the lipid structure of liposomes animates the combination of autophagic layers, which might be helpful for advancing autophagy. Nonetheless, apparently, they are not ideal for the course of autophagy concealment. The study by Gao et al. looked into the link between autophagy and polyethylenimine-triggered cytotoxicity in MDCK (Madin-Darby canine kidney) and Chang liver cell lines [185]. They presumed that autophagy assumes a significant role in the improvement of PEI's cytotoxicity, as they could show that, although animating autophagy upgrades the speed at which cells bite the dust, impeding autophagy diminishes the cytotoxicity delivered by PEI. Another finding showed that the damage caused by PEI-mediated autophagy happens in two stages: (1) an initial stage that lasts 3 h and hurts lysosomes; and (2) a later stage that lasts 24 h and hurts mitochondria. Another review found that [186]. They showed that while enacting autophagy improves the rate at which cells pass on, stifling autophagy diminishes the cytotoxicity produced by PEI, driving them to presume that autophagy assumes a critical role in the expansion of PEI's cytotoxicity. They reached the decision that autophagy is a vital component. The research has also shown that the cytotoxicity brought on by PEI-mediated autophagy occurs in two stages: (1) an early stage that lasts for 3 h and is responsible for causing damage to lysosomes, and (2) a later stage that lasts for 24 h and is responsible for causing damage to mitochondria. Another piece of investigation discovered that [187]. It would suggest that the aggregation of NPs after their internalization by the cell has a detrimental effect on autophagy [188]. It is possible for the anti-inflammatory function of nanocarriers to be mediated through their modulatory influence on autophagy. Upconversion NPs that encapsulate chlorin e6 efficiently promote the creation of reactive oxygen species (ROS). This, in turn, stimulates autophagy via the PI3K/Akt/mTOR signaling pathway, which ultimately results in a down-regulation of the production of pro-inflammatory cytokines (IL-12, TNF-β, and iNOS). When all is considered, nanoparticles have the ability to control autophagy, and this capability has the potential to be used in medicinal applications in a way that holds great promise. Nevertheless, these nanoparticles have the potential to cause toxicity in other organs, particularly those that are closely associated with autophagy. For example, exposure to ZnO NPs may have negative effects on the gastrointestinal system; however, the induction of autophagy is being investigated as a possible method for reducing the severity of these toxic side effects [189]. Recent research has highlighted another crucial role of nanocarriers: their modulation of autophagy, a cellular process with implications for various diseases [190,191]. For instance, nanoparticles can induce autophagy by triggering cellular stress responses or by directly interacting with autophagic machinery [192,193]. On the other hand, some nanocarriers may inhibit autophagy, which can be beneficial in specific disease contexts [194].

Challenges in Designing Drug Delivery Systems Considering Autophagy Modulation.

### **8.1** Selective autophagy modulation

Achieving precise control over autophagy is challenging. Nanocarriers must be designed to target specific cell types and deliver cargo to autophagosomes selectively [195].

### 8.2 Autophagy regulation in disease context

The impact of autophagy modulation by nanocarriers may vary depending on the disease being targeted. Understanding disease-specific autophagy alterations is crucial [196].

### 8.3 Safety and biocompatibility

Ensuring the safety and biocompatibility of nanocarriers is essential to avoid unintended side effects, including excessive autophagy induction or inhibition [197].

### 8.4 Cargo delivery efficiency

Nanocarriers must efficiently deliver therapeutic cargo to the target site while avoiding premature cargo release or degradation [198].

### 8.5 Clinical translation

Getting nanocarrier-based autophagy modulators from preclinical research to clinical use means getting past concerns about regulation and production [199]. Recent research has made significant strides in addressing the challenges mentioned above. Advanced nanocarrier formulations, such as stimuli-responsive nanoparticles and targeted liposomes, have been developed for precise autophagy modulation [200,201]. Additionally, nanocarrier-based combination therapies that leverage autophagy modulation have shown promise in preclinical studies [202].

Designing drug delivery systems that consider nanocarriers' role as autophagy modulators presents a promising avenue for improving the treatment of various diseases. However, several challenges must be addressed to harness the full potential of nanocarriers in this context. Recent advances in nanocarrier technology and our understanding of autophagy regulation are paving the way for innovative therapeutic strategies. Further research and collaboration between disciplines such as nanotechnology, pharmacology, and cell biology are needed to overcome these challenges and bring nanocarrier-based autophagy modulators to the clinic. It is important to point out that nanoparticles have the ability to either stimulate or suppress autophagic cell fates [[203], [204]]. As a result, autophagy nano-inhibitors should not be used for the transport of autophagy inducers, while autophagy nano-inducers are not viable candidates for use as nanocarriers for autophagy inhibitors.

## 9 Trafficking and nano-delivery in mammalian cell biology

In mammalian cell biology, trafficking is integral to the function of nano-delivery systems, guiding the transport of nanoparticles within the cellular landscape. These systems use endocytosis and other cellular pathways to get inside cells and then find their way to certain organelles or cell compartments [205]. Nanoparticles are often made with changes to their surfaces that make them easier for cells to take in and help them find their way inside, which improves therapy delivery and lowers systemic side effects. This level of accuracy in nano-delivery is essential for treatments that target specific parts of cells, like the nucleus or mitochondria, making sure that therapeutic agents work better and cause less harm [206].

Cellular trafficking is important for nano-delivery systems to work in mammalian cells because it controls what happens to nanoparticles inside cells. Upon entering the cell, nanoparticles undergo a series of transport processes, moving through various compartments, such as endosomes and lysosomes. This intracellular journey is vital for the release and bioavailability of the therapeutic payload at the target site. Advanced nanoparticle designs incorporate ligands for receptor-mediated endocytosis, enhancing specificity and efficiency [207]. Understanding these trafficking pathways allows for the optimization of nanoparticle design, maximizing therapeutic efficacy while minimizing unintended effects. Research continues to unravel these complex interactions, aiming to harness cellular trafficking mechanisms to improve drug delivery and response in clinical settings [208].

Nanoparticle trafficking within mammalian cells is a sophisticated process that determines the effectiveness of nano-delivery systems. After cellular entry, typically via endocytosis, nanoparticles are routed through various organelles, influencing their release and the bioavailability of their cargo. Nanoparticle design innovations, such as changing the surface and using targeting ligands, aim to improve targeting to specific sites like mitochondria or the nucleus, which should lead to better therapeutic outcomes [209]. Researchers are still looking into these cellular processes to make nanoparticle-based therapies better. This fits with how medical nanotechnology is changing and opens up new possibilities for targeted treatment strategies [210].

## 10 Autophagy and trafficking in mammalian cells

Autophagy and trafficking are interconnected processes crucial for maintaining cellular homeostasis in mammalian cells. Autophagy, a mechanism for degrading and recycling cellular components, collaborates with the cell's trafficking system to ensure proper cargo delivery to lysosomes. This synergy is vital for nutrient regulation, organelle quality control, and the elimination of pathogens [211]. Disruptions in the coordination between autophagy and trafficking can lead to various diseases, including neurodegeneration and cancer. Understanding these pathways offers insights into their roles in health and disease, providing potential therapeutic targets [212].

In mammalian cells, the interplay between autophagy and trafficking is pivotal for cellular function and health. Autophagy not only degrades and recycles cellular components but also influences trafficking by directing cargo to lysosomes. This coordination is essential for various cellular processes, including immune responses and protein quality control [213]. Disruptions in these pathways can contribute to diseases like cancer and neurodegeneration, highlighting their importance in cell biology and medicine. Research in this area continues to uncover the complex mechanisms and potential therapeutic implications of autophagy and trafficking [214].

Autophagy and trafficking in mammalian cells are essential for maintaining cellular integrity, with their interplay being critical for various physiological processes. The autophagic machinery works closely with cellular trafficking systems to ensure efficient degradation and recycling of cellular components [215]. This collaboration is vital for the regulation of cellular metabolism, defense against pathogens, and the removal of damaged organelles. Autophagy and trafficking are two important processes that need to be carefully balanced in order for cells to stay healthy. Researchers are looking into this balance and are looking for ways to treat diseases where these processes are out of whack [216].

## 11 Autophagy and endosomal pathway

The interplay between autophagy and the endosomal pathway is crucial in mammalian cells, facilitating the degradation and recycling of cellular components. Autophagy intersects with the endosomal system at various stages, including the formation of amphisomes, where autophagosomes merge with endosomes. This convergence is essential for the lysosomal degradation of intracellular materials [217]. Figuring out how autophagy and the endosomal pathway talk to each other is important for learning about how cells stay healthy, how signals work, and how diseases start, as it could lead to new treatments [218].

The relationship between autophagy and the endosomal pathway in mammalian cells is intricate and vital for cellular function. The endosomal system works with autophagic processes. One way this happens is through the creation of amphisomes, which are formed when autophagosomes and endosomes join together before breaking down in lysosomes. This interplay is crucial for regulating cellular responses to stress, nutrient availability, and pathogen invasion [219]. Breaking down how autophagy and endosomal pathway crosstalk work can help us understand how cells stay healthy and how diseases work, which could lead to new ways to treat them [220].

The interplay between autophagy and the endosomal pathway is a key aspect of cellular function, impacting processes like nutrient recycling, immune responses, and cellular signaling. The formation of amphisomes, resulting from the fusion of autophagosomes with endosomes, underscores the intricate coordination between these pathways, which is essential for the lysosomal degradation of cellular components [221]. This crosstalk is not only pivotal for maintaining cellular homeostasis but also offers insights into the mechanisms underlying various diseases, highlighting its potential as a target for therapeutic interventions [222].

## 12 Strategy to target autophagy for regulation of mammalian cellular trafficking for better nano-delivery

Targeting autophagy to regulate mammalian cellular trafficking for enhanced nanodelivery involves manipulating autophagic pathways to optimize the internalization and intracellular routing of nanoparticles. Strategies include designing nanoparticles that can modulate autophagic flux, thereby influencing their uptake and degradation [223]. This approach ensures the targeted delivery of nanoparticles to specific cellular compartments, enhancing the therapeutic efficacy of the delivered agents. Researchers can make advanced nano-delivery systems that use autophagic pathways to improve drug delivery and treatment outcomes [224] by learning more about how autophagy works at the molecular level and how it affects the movement of cells.

Enhancing nano-delivery through autophagy involves sophisticated strategies that modulate autophagic pathways, improving nanoparticle trafficking and delivery. This includes the development of nanoparticles that can either induce or inhibit autophagy, tailored to the specific therapeutic context [225]. For example, nanoparticles designed to evade autophagic degradation can enhance drug delivery to target sites, while those that promote autophagic processes can assist in the clearance of pathogenic substances within cells. Understanding the nuanced interactions between autophagy and nanoparticle trafficking is key to designing more effective therapeutic interventions, with the potential to revolutionize treatments for a variety of diseases [226].

To further enhance nanodelivery via autophagy modulation, researchers are exploring advanced strategies that involve designing nanoparticles to specifically interact with key components of the autophagic machinery. One way to do this is to target genes or proteins related to autophagy to change the autophagic process and make it easier for cells to take nanoparticles up and spread them out [227]. Additionally, the development of stimulus-responsive nanoparticles that can respond to autophagic signals offers a dynamic approach to controlling the release and activity of therapeutic agents within cells. Such strategies are at the forefront of merging nanotechnology with cellular biology, offering promising avenues for targeted and efficient drug delivery systems with the potential to significantly improve patient outcomes [228].

To make the field even better, researchers are looking into the complicated link between autophagy and nanoparticle trafficking. They are making nanoparticles that can change the way autophagic pathways work. This includes making nanoparticles that can interact with certain autophagy-related proteins or that are sensitive to the autophagic environment. This makes them more effective at getting into cells and treating diseases [229]. Using new methods, like autophagy-inducing peptides or small molecules attached to nanoparticles, to precisely control autophagy and provide a personalized therapeutic response is one goal. These developments show how autophagy modulation and nanotechnology can be used together to make very specific and effective drug delivery systems. This could lead to new ways of treating complicated diseases [230].

To learn more about how autophagy and nanoparticle trafficking work together, scientists are now trying to find the autophagic regulators and signaling pathways that nanoparticles can interact with. To do this, scientists have to make nanoparticles that can either stop or slow down these pathways, which changes the cellular fate of their therapeutic payload [231]. The incorporation of autophagy-targeting ligands into nanoparticles presents a method to enhance specificity and reduce off-target effects, aligning drug delivery with cellular metabolic states. Such cutting-edge approaches exemplify the dynamic interplay between nanotechnology and cell biology, aiming to refine therapeutic strategies and improve clinical outcomes in diseases where autophagy plays a key role [232].

## 13 Organelle autophagy relation to specific organelle targeting via selective autophagy and targeting systems

Organelle autophagy, which includes mitophagy, reticulophagy, and lipophagy, are specific autophagic processes that break down and recycle different organelles. Mitophagy selectively degrades damaged mitochondria, which is crucial for cellular energy homeostasis and preventing apoptosis. Reticulophagy involves the selective autophagy of the endoplasmic reticulum, playing a key role in protein homeostasis [233]. Lipophagy targets lipid droplets, influencing lipid metabolism and energy balance. Using selective autophagy to connect these processes to targeted therapeutic strategies can improve the efficiency and specificity of drug delivery, which could lead to better ways to treat diseases affecting organelles [234].

Mitophagy is essential for eliminating defective mitochondria, thereby preventing cellular stress and promoting metabolic efficiency. Reticulophagy ensures the turnover of endoplasmic reticulum fragments, maintaining protein folding and processing fidelity. Lipophagy regulates lipid stores, influencing energy production and lipid homeostasis [235]. More and more people are realizing that these selective autophagic processes can be used as therapeutic targets. By changing how certain organelles break down, doctors can change how diseases progress. Nanotechnology has made it possible for targeted delivery systems to change these autophagic pathways. This opens up new ways for precision medicine to treat disorders that affect organelles [236].

To further explore organelle-specific autophagy, it's crucial to delve into additional mechanisms and implications. For instance, pexophagy, the selective degradation of peroxisomes, is vital for cellular detoxification processes. Similarly, nucleophagy, involving the selective autophagy of nuclear components, plays a role in nuclear quality control and genome stability [237]. Targeted intervention in these selective autophagic pathways could fix organelle dysfunction in diseases, making them useful for therapeutic strategies. New developments in drug delivery systems, like organelle-targeted nanoparticles, make it possible to change these pathways in a way that could lead to new treatments for cancer, metabolic disorders, and neurodegenerative diseases [238].

We delve into xenophagy, the autophagic degradation of invading pathogens, showcasing autophagy's role in cellular defense. Glycophagy, another variant, involves the selective degradation of glycogen, illustrating autophagy's metabolic significance. These specialized forms of autophagy highlight the pathway's adaptability and specificity for cellular maintenance and defense [239]. Advanced drug delivery systems, like organelle-targeted nanoparticles, make it possible to target these specific autophagic processes. This opens up new therapeutic options and provides targeted approaches for diseases involving organelle dysfunction [240]. To learn more, chaperone-mediated autophagy (CMA) is a type of selective autophagy in which certain proteins are moved directly across the lysosomal membrane to be broken down. This process is crucial for protein quality control and cellular homeostasis [241]. Understanding and targeting such specific autophagy pathways can lead to innovative therapeutic approaches, particularly in age-related diseases where autophagy efficiency declines. New developments in nanotechnology make it possible to change these autophagy pathways selectively. This opens up new ways to use precision medicine to treat a wide range of diseases that are linked to organelle and cell dysfunction [242].

It's essential to consider how different types of selective autophagy, beyond the degradation of organelles, play roles in cellular health and disease. For instance, ferritinophagy, the selective autophagy of ferritin, regulates iron homeostasis, which is crucial for many cellular processes [243]. By targeting these specific autophagy pathways, researchers can develop targeted therapies that address the underlying causes of various diseases, such as neurodegeneration, where iron accumulation is a factor. Nanotechnology and other advanced drug delivery systems could be used to change these selective autophagy pathways. This would open up new ways to treat diseases and improve precision medicine [244]. One part of the complex relationship between selective autophagy and organelle health is lysophagy, which breaks down damaged lysosomes in a certain way. This process is vital for cellular homeostasis and prevents the leakage of lysosomal enzymes into the cytoplasm, which can cause cell death [245]. Using lysophagy and other specific autophagic pathways as targets can help treat diseases where lysosomes don't work right. The creation of targeted nanocarriers that can change these pathways is a promising way to make treatments more specific and effective for a wide range of metabolic and degenerative diseases [246].

In the case of nanoparticles and drug delivery vectors, combining nanotechnology with selective autophagy pathways means we can deliver drugs more precisely. Nanoparticles can be designed to change certain autophagic processes, which makes therapeutic delivery more precise and effective [247]. For instance, nanoparticles designed to target lysophagy could improve the delivery of drugs to lysosome-related disease sites. This approach not only enhances the therapeutic index of drugs but also minimizes off-target effects, representing a significant advancement in the field of targeted therapy and precision medicine [248]. To further the integration of nanotechnology with selective autophagy for drug delivery, researchers are exploring nanoparticles that can specifically target and modulate autophagic pathways associated with different organelles. Such targeted nanoparticles can enhance the delivery and efficacy of therapeutics by ensuring their release in the vicinity of or directly within the targeted organelles, optimizing drug action, and minimizing systemic toxicity [249]. This accuracy in aiming at autophagy pathways specific to organelles is a big step forward in the creation of highly targeted nanomedicine strategies. It could lead to new ways of treating a wide range of diseases, including those where organelle dysfunction is a major factor [250].

When looking at the connection between nanoparticles, drug delivery vectors, and autophagy, the focus now shifts to making nanoparticles that can interact with certain autophagic processes to improve targeted therapy. Nanoparticles can be made to either start or stop autophagy in specific cells, which changes what happens to the drugs inside the cells [251]. This targeted approach can significantly improve the therapeutic index of drugs, especially in diseases where autophagy plays a key role in pathogenesis or therapy resistance. Such advancements in nanotechnology and autophagy research hold the promise of developing more effective and less toxic therapeutic strategies, potentially revolutionizing the treatment of complex diseases [252]. To learn more about how nanoparticles, drug delivery vectors, and autophagy work together, it's important to think about how to make nanoparticles that can precisely change autophagic pathways in specific cells or organelles [253]. Such designs can significantly enhance therapeutic outcomes by ensuring drug delivery is synchronized with the cell's autophagic state, potentially overcoming drug resistance mechanisms or improving the delivery of therapeutics to hard-to-reach cellular locales. As this field continues to grow, it will likely lead to new ways of treating diseases, especially cancer and neurodegenerative diseases, where changing autophagy can be very important for effective treatment [254].

Exploration into the intersection of nanoparticles, drug delivery vectors, and autophagy unveils potential advancements in precision medicine. Nanoparticles can be tailored to selectively modulate autophagic activity in specific cell types or organelles, enhancing drug delivery and therapeutic efficacy [255]. This approach could revolutionize treatments for diseases where autophagy plays a critical role, such as in targeted cancer therapy or neurodegeneration, by facilitating targeted drug release and reducing off-target effects, ultimately leading to more effective and personalized treatment strategies [256].

Building on the work that has already been done to combine nanoparticles with autophagy for drug delivery, scientists are now looking into how to make nanoparticles respond to the autophagic state of cells so that drugs can be released or activated only at certain sites. This strategy has the potential to enhance the selectivity and effectiveness of therapeutics, particularly in scenarios where autophagy's modulation can directly influence treatment outcomes [257]. Such innovative approaches in nanoparticle design could lead to groundbreaking advancements in targeted therapies for a range of diseases, offering a new paradigm in the nexus of nanotechnology, autophagy, and personalized medicine [258]. The development of nanoparticles that interact with autophagic pathways can enable more nuanced control over drug delivery mechanisms, tailoring therapies to the unique cellular environments of different diseases. For instance, in cancer therapy, nanoparticles that modulate autophagy can either enhance the cytotoxicity of chemotherapeutic agents or protect normal cells from undesired side effects [259]. Similarly, in neurodegenerative diseases, where autophagic balance is crucial, nanoparticles can be designed to restore cellular homeostasis. These advancements underscore the potential of combining nanotechnology with autophagy to create more precise and effective therapeutic interventions [260].

The synergy between nanoparticles and autophagy and the potential for targeted therapy are immense. Nanoparticles can be engineered to deliver drugs that specifically activate or inhibit autophagy in diseased cells, optimizing therapeutic outcomes [261]. This approach is particularly promising in areas like targeted cancer therapy, where autophagy modulation can sensitize tumour cells to chemotherapeutics, and in neurology, where correcting autophagy imbalances could halt disease progression. Such targeted strategies signify a leap toward personalized medicine, leveraging the precision of nanotechnology to modulate cellular processes in disease-specific contexts [262].

## 14 Secretory autophagy, exetra cellular vesicles, and nano-delivery

Secretory autophagy is different from normal degradative autophagy. It moves things from the cytoplasm to the extracellular space, and extracellular vesicles (EVs) help with this. This pathway has significant implications for intercellular communication and can be exploited for targeted nanodelivery [263]. Nanoparticles can be made to improve or copy the secretory autophagy pathway, which makes it easier for therapeutic agents to get to cells through EVs. This approach could improve the specificity and efficiency of drug delivery, offering new avenues for the treatment of various diseases by leveraging the natural cellular processes of secretion and intercellular signaling [264]. It's evident that this interplay offers innovative strategies for drug delivery. Using the secretory autophagy pathway, nanoparticles can be made to attach to or be enclosed by EVs, which makes drug delivery more targeted and effective [265]. This method leverages natural cellular processes for enhanced bioavailability and intercellular communication, potentially reducing side effects and improving therapeutic outcomes. Such advancements in nanoparticle design and application could revolutionize treatments in various medical fields, aligning with the goals of personalized medicine and targeted therapy [266].

Using secretory autophagy for nanoparticle-based drug delivery means using EVs as natural carriers, which makes therapeutic delivery more precise and effective. By incorporating nanoparticles into EVs, which are integral to secretory autophagy, drugs can be directed more effectively to specific target cells, utilizing the body's innate signaling pathways [267]. This method not only promises increased targeting accuracy but also the potential for reduced systemic toxicity, aligning with the objectives of precision medicine. Innovations in this area could lead to breakthroughs in how treatments are delivered, offering new solutions for a range of diseases [268]. Delving deeper into the nexus of secretory autophagy and drug delivery, we can explore how this integration can be optimized for therapeutic innovation. Creating nanoparticles that can use or copy the secretory autophagy pathway to make it easier for drugs to get inside EVs is a big step forward in targeted therapy [269]. This approach can significantly enhance drug delivery specificity, minimize off-target effects, and maximize therapeutic efficacy. Such advancements hold the promise of revolutionizing the delivery of a wide array of therapeutics, from small-molecule drugs to biologics, providing a platform for more personalized and precise medical interventions [270].

Further exploration delves into the potential of creating a symbiotic relationship between nanotechnology and cellular processes. Scientists can use the natural ability of secretory autophagy to target and route cells by making nanoparticles that can selectively attach to or encapsulate EVs [271]. This approach could completely change how medicines are delivered and how well they work, especially when it comes to reaching hard-to-reach targets or getting past biological barriers. It would add a new level of accuracy to nanoparticle-mediated drug delivery systems [272].

Building on the synergy between nanoparticles, secretory autophagy, and EVs in drug delivery, further advancements can be envisioned in the customization of nanoparticles to exploit the secretory pathways of cells [273]. We can make it easier for drugs to get to the right cells or tissues by designing nanoparticles that work with the secretory autophagy process. This lowers the amount of drugs that get into the whole body and improves the effectiveness of therapy. This approach could lead to significant innovations in the way treatments are administered, particularly for diseases where targeted delivery is crucial, offering a new layer of specificity and efficiency in drug delivery mechanisms [274]. The integration of nanoparticles with secretory autophagy and EVs presents a novel paradigm in drug delivery vectors, enabling more targeted and efficient therapeutic interventions [275]. By tailoring nanoparticles to interact with or be encapsulated by EVs, it's possible to harness the body's natural cellular processes for enhanced drug delivery. This approach can improve the specificity of drug targeting, reduce off-target effects, and potentially overcome biological barriers, offering promising new avenues for the treatment of various diseases [276].

## 15 Conclusion

As we just talked about, the way the fats are organized in liposomes makes the joining of autophagic membranes stronger, which could help speed up autophagy. Nonetheless, apparently, they are not ideal for the course of autophagy concealment. The study by Gao et al. looked into the link between autophagy and polyethylenimine-induced cytotoxicity in MDCK (Madin-Darby canine kidney) and Chang liver cell lines [185]. They presumed that autophagy assumes a significant role in the upgrade of PEI's cytotoxicity, as they could show that, although animating autophagy improves the speed at which cells kick the bucket, impeding autophagy diminishes the cytotoxicity delivered by PEI. This study also showed that the damage caused by PEI-mediated autophagy happens in two stages: (1) an initial stage that lasts 3 h and hurts lysosomes; and (2) a later stage that lasts 24 h and hurts mitochondria. Another review found that [186] They exhibited that while enacting autophagy improves the rate at which cells bite the dust, smothering autophagy diminishes the cytotoxicity created by PEI, driving them to presume that autophagy assumes a critical role in the expansion of PEI's cytotoxicity. They reached the determination that autophagy is a significant component [277,[278], [279], [280]].

Autophagy may be either selective, in which it only degrades certain compounds, or non-selective, in which it degrades many materials regardless of the nature of those materials. It can maintain homeostasis while also generating illness, which means that it can have an effect on both health and disease since it can play both physiological and pathological functions. When autophagy comes into contact with viral and non-infectious disorders, it may either act as a protective mechanism or make the condition worse. Autophagy has twin purposes, one of which is to inhibit the growth of bacterial, viral, and tumorous infections, while the other is to promote the advancement of tumours, bacterial infections, and viral infections. Autophagy is a process that may either support healthy brain growth, immunity, and cardiovascular and endocrine development, or it can lead to neurodegeneration, autoimmune illnesses, cardiovascular diseases, obesity, and diabetes. Its dual functions in physiology and pathology give it the ability to play both of these roles.

It has been discovered that highly developed nanoparticles have the potential to serve as intelligent drug delivery systems. These systems might enhance the therapeutic impact of already-available conventional medications and improve patient survival rates. There is no question that there is still a significant distance to go between the creation of nanocarriers and their introduction as viable items on the pharmaceutical market. Although hundreds of research publications report fantastic results of drug delivery systems with varied natures and qualities in many in vitro and in vivo cancer models, only a tiny proportion have successfully reached trials for their usage in the clinic. This is due to the fact that only a small fraction of drug delivery systems have been shown to be effective. The inadequate therapeutic effectiveness and off-target toxicity in key organs are mostly to blame for the poor clinical translation of novel nanoparticles.

## Data availability

No data was used for the research described in the article.

## CRediT authorship contribution statement

**Moataz Dowaidar:** Writing – review & editing, Writing – original draft, Formal analysis, Data curation, Conceptualization.

## Declaration of competing interest

The authors declare that they have no known competing financial interests or personal relationships that could have appeared to influence the work reported in this paper.
